# Hirsutism, Normal Androgens and Diagnosis of PCOS

**DOI:** 10.3390/diagnostics12081922

**Published:** 2022-08-09

**Authors:** Poli Mara Spritzer, Lucas Bandeira Marchesan, Betânia Rodrigues Santos, Tayane Muniz Fighera

**Affiliations:** 1 Gynecological Endocrinology Unit, Division of Endocrinology, Hospital de Clínicas de Porto Alegre, Porto Alegre 90035-903, RS, Brazil; 2 Post-Graduate Program in Endocrinology, Medicine School, Universidade Federal do Rio Grande do Sul, Porto Alegre 90035-003, RS, Brazil; 3 Department and Post-Graduate Program in Physiology, Universidade Federal do Rio Grande do Sul, Porto Alegre 90035-003, RS, Brazil; 4 Department of Internal Medicine, Universidade Federal do Rio Grande do Sul, Porto Alegre 90035-003, RS, Brazil

**Keywords:** polycystic ovary syndrome, biochemical hyperandrogenism, Ferriman–Gallwey score, hirsutism

## Abstract

Hirsutism is defined as the presence of terminal hair with male pattern distribution in women. While in the general population, hirsutism affects around 4–11% of women, it is the main manifestation of hyperandrogenism in women with polycystic ovary syndrome (PCOS), with a prevalence estimated at 65–75%. Hirsutism in PCOS is associated with both androgen excess and individual response of the pilosebaceous unit to androgens. The modified Ferriman–Gallwey (mFG) scoring system has been widely used in clinical practice to visually score excessive terminal hair, thus standardizing hirsutism evaluation and facilitating data comparison. Although a universal mFG score cutoff would be useful for comparisons, ethnic variations, as well as skin type and other factors, should be considered when evaluating hirsutism in distinct populations. In turn, androgen levels, measured by conventional techniques, have been shown to correlate poorly with the severity of hirsutism. Indeed, while most women with PCOS and hirsutism also have higher than reference values for serum androgen levels, some of them may not present with biochemical hyperandrogenism, representing a challenge to the diagnosis of PCOS. In this article, we critically review this not uncommon condition in women with PCOS presenting with hirsutism but normal androgen levels.

## 1. Introduction

Hirsutism is defined as an abnormal amount of terminal hair in a male pattern distribution in women and is the main manifestation of hyperandrogenism in women with polycystic ovary syndrome (PCOS). Acne and female pattern hair loss (FPHL) are also clinical manifestations of hyperandrogenism, although mild acne and FPHL are not recommended as diagnostic criteria in adolescents due to the paucity of data in this population, with progressive hirsutism remaining the primary marker of hyperandrogenism [1].

In response to increased androgen levels at puberty, vellus hair follicles in specific areas develop into terminal hair (larger, curlier, and darker, hence more visible), becoming sexual-hair follicles [2]. While pubic and axillary hair are quite sensitive to small amounts of androgens, other areas may need higher androgen concentration for follicle terminalization [3]. Free testosterone is the main active portion of plasma testosterone and is responsible for this action [4]. 

The broad and heterogeneous clinical expression of PCOS gave rise to the perception that no single criterion should be mandatory for the diagnosis of PCOS, which resulted in the 2003 Rotterdam diagnostic criteria. According to that consensus, PCOS should be diagnosed when at least two of the following criteria are present: ovulatory dysfunction (oligo- or amenorrhea), hyperandrogenism (either biochemical or clinical), and polycystic ovarian morphology (follicle number per ovary of ≥20 and/or an ovarian volume ≥ 10 mL on either ovary, preferably with an endovaginal ultrasound) [1,5,6], excluding other related or mimicking disorders. The Rotterdam criteria for defining PCOS were endorsed by the US National Institutes of Health (NIH) in 2012 (https://prevention.nih.gov/research-priorities/research-needs-and-gaps/pathways-prevention/evidence-based-methodology-workshop-polycystic-ovary-syndrome-pcos (accessed on 21 June 2022)) and by the International Guideline on PCOS in 2018, based on the best available evidence [1] (Expert Panel from a NIH Evidence-Based Methodology Workshop on PCOS, December 2012). Indeed, while most women with PCOS and hirsutism also have higher than the reference values for serum androgen levels, some of them may not present with biochemical hyperandrogenism, representing a challenge for the diagnosis of PCOS. In this article, we critically review this not uncommon condition in women with PCOS presenting with hirsutism but normal androgen levels.

## 2. Hirsutism and Hair Follicle Cycling

Hirsutism is defined as the presence of terminal hair of the female body in male pattern. The male sexual pattern is observed in androgen-sensitive anatomic sites that include the face, chest, breast areola, linea alba, lower back, buttocks, inner thighs, and external genitalia [7,8]. While in the general population, hirsutism affects 4–11% of women, in PCOS, its prevalence is estimated at 65–75% [9] and severity varies according to the degree of androgen excess and individual variability in the sensitivity of the pilosebaceous unit to androgens [10,11]. 

Hair follicles are small organs formed by the interaction between epidermis and dermis and are important skin appendages. They exhibit a periodic growth cycle that occurs continuously throughout the human lifespan and have high regenerative capacity. These characteristics are due to the presence of many stem cell populations in the hair follicle, and the activity of these stem cells and growth of hair follicles are highly regulated by various signaling pathways [12]. Moreover, hair growth is affected by many factors, such as age, environment, and health status, which can influence the development of hair follicle tumors, alopecia areata, and other related diseases, such as hirsutism [13].

In humans, there are three types of hair: lanugo, vellus, and terminal hair. Lanugo is the very fine hair that covers the fetal body and disappears during the first weeks of life. Vellus hair is very short, fine, and usually non-pigmented, whereas terminal hair is longer, thick, and pigmented. In women, terminal hair is found mostly in such areas as the eyebrows, eyelashes, scalp, axilla, and pubis. Less commonly, women may also have terminal hair in areas of the so-called male sexual pattern. Hormonal influence may alter the pattern of hair distribution in women, leading to excessive hair growth [11,13].

The hair follicle cycle is divided into three stages named anagen, catagen, and telogen, which characterize the perpetual cycle of growth, involution, and rest, respectively. The anagen stage is the most active period of hair follicle growth, when the hair grows rapidly and forms a complete hair shaft. Epithelial cell differentiation and hair pigmentation occur during this stage. The duration of the anagen stage determines hair length and is associated with the continuous proliferation and differentiation of stromal cells at the base of hair follicles [11,13,14]. Moreover, there is substantial variation in anagen duration according to body region: up to 6 years in the scalp, 1 to 3 months in the arms, 4 to 6 months in the legs, and 1 to 2 months in the thighs. Approximately 85% to 90% of all scalp hair is in the anagen stage at any given moment. This figure changes according to the body region, age, and possibly gender. In the arms and legs, approximately 46% and 58% of all hair, respectively, is always in the anagen stage [11]. When hair follicles enter the catagen stage, hair usually stops growing and, at the end of this phase, the hair follicles have atrophied. Therefore, the catagen stage starts when the supply of matrix cells declines, leading to a slowdown in the differentiation of the hair shaft and the inner root sheath (apoptosis-driven regression) [13,14]. During catagen, hair follicle degeneration is highly regulated, and a large number of keratinocytes from the hair follicle begin to undergo programmed death. At this stage, melanin production in hair follicles stops and melanin-containing cells in some hair follicles also begin to undergo apoptosis [13]. After regression, the hair follicle enters the telogen stage, recognized as a resting phase. This stage is characterized by low biological activity of the follicle and a drop in hair shaft. Telogen duration changes according to body region: 2 to 4 months in the scalp, 2 and a half months in the chest, 2 to 4 months in the arms, and 3 to 6 months in the legs [11,13,14]. The follicle remains in the telogen stage until it is reactivated. At this point, the expression and activity of the relevant regulatory factors in the hair follicle controlling its cyclical growth will be significantly enhanced to prepare for the beginning of the next anagen [11,13,14].

## 3. Actions and Metabolism of Androgens in the Hair Follicle

The importance of androgens for hair growth in humans was first established by Hamilton and colleagues in the 1950s [15], with observations that castration before puberty prevented the development of beard and axillary hair, whereas castration after puberty led to the atrophy of beard and axillary hair. In addition, patients with androgen insensitivity do not have pubic or axillary hair. Since then, androgens have been shown to increase hair follicle size in different body regions, hair diameter, and the proportion of time hair remains in the anagen stage [16]. The conversion of vellus to terminal hairs is mainly stimulated by androgens, through the prolongation of the anagen stage. Successive hair cycles and longer anagen duration promote an increase in follicle size. These larger follicles produce longer, thicker hair in androgen-dependent body areas. For this reason, androgen excess-related conditions, such as PCOS, are often associated with hirsutism [11,17].

It is important to note that the occurrence and severity of hirsutism are also associated with the sensitivity of hair follicles to androgens. In fact, not only do hair follicles respond to androgens, broadly expressing androgen receptors, but they also contain androgen-metabolizing enzymes that play a critical role in regulating the level of androgens in the hair follicle [11,17,18]. Main enzymes include cytochrome P450 aromatase, which converts testosterone and androstenedione to estrogens (17β-estradiol and estrone, respectively), type 2 17β-hydroxysteroid dehydrogenase (17β-HSD), which inactivates testosterone to androstenedione, and 5α-reductase, which converts testosterone to dihydrotestosterone (DHT). Therefore, altered expression of these enzymes may be associated with a greater or lesser androgenic activity in the hair follicle.

**Figure 1:**
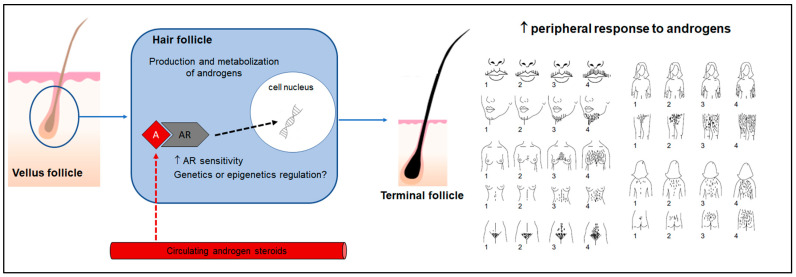
Role of androgens in the hair follicle and its peripheral response. A: androgens; AR: androgen receptor; ↑ peripheral response to androgens is expressed graphically by the modified Ferriman–Gallwey scale.

Previous studies have shown that the expression of the type 2 17β-HSD gene is lower in scalp hair follicles of women with hirsutism than of women without it but similar to that of men [19], and higher in the subumbilical region and arm skin [20]. These results suggest that type 2 17β-HSD may play a role in the development of hirsutism, as the enzyme is responsible for inactivating more potent sex steroids (such as testosterone) by oxidation reactions [19]. Other gene expression analyses using 5α-reductase confirmed the presence of isotype 1 on the vertex of the scalp in women, but there was no significant difference in the expression patterns of women with or without hirsutism [21]. No correlation was found between 5α-reductase type 1 and 2 gene expression in dermal papillae from the lower abdominal region and severity of hirsutism [22]. In turn, variants in the gene encoding 5α-reductase type 1 were associated with hirsutism in women with PCOS [23]. In previous studies, we found no associations between 17β-HSD or aromatase gene variants and the severity of hirsutism in women with PCOS [24,25] (Figure 1).

## 4. Molecular and Genetic Regulation of Hair Follicle

Different molecular signaling pathways are involved in the control of hair follicle growth and cycling [13]. MicroRNAs and gene variants in the androgen receptor gene appear to be promising for understanding the pathophysiology of hirsutism. MicroRNAs can contribute to the regulation of hair follicle morphogenesis and regeneration [13,14]. Shorter CAG repeat polymorphisms of the androgen receptor gene may be associated with increased activity of the receptor, leading to PCOS and hirsutism [26]. However, while some studies have shown a negative association between CAG repeat lengths and the prevalence of hyperandrogenic states, such as PCOS [27,28,29,30,31], or more severe clinical hyperandrogenism, such as hirsutism in women with PCOS [32], meta-analyses of studies correlating CAG repeat lengths with PCOS [33,34] and data in hirsute women with and without hyperandrogenemia [35,36] do not confirm this association. Therefore, many questions remain unanswered regarding the precise mechanisms underlying androgen/androgen receptor signaling pathways. 

## 5. Scoring Hirsutism in the Context of PCOS

Although hirsutism reflects androgen action in the hair follicle, local factors, sensitivity to androgen, duration of exposure and local conversion of testosterone to a more potent androgen, DHT, by 5α-reductase may be more important than plasma androgen levels [37,38]. Among local factors, an important contributor to hair follicle development and growth is the activity of L-ornithine decarboxylase, an enzyme that catalyzes the synthesis of polyamines implicated in cell migration, proliferation and differentiation of hair follicles, androgen receptor concentration, and 17β-HSD and 5α-reductase activities [3]. Most women with androgen levels more than twice the upper limit of the reference range have some degree of hirsutism, but it has been demonstrated that androgen levels, measured by conventional techniques, correlate poorly with the severity of hirsutism [39]. Likewise, women may present with excessive body hair growth but normal plasma androgens levels. Because of these observations, the most used criteria for the diagnosis of PCOS consider the hyperandrogenism criterion as clinical and/or biochemical [1]. Clinical hyperandrogenism is still very important while defining hyperandrogenism for the PCOS diagnosis, especially in women with normal androgens. 

The Ferriman–Gallwey (FG) scoring system has been widely used in clinical practice to visually score excessive terminal hair [40], thus standardizing hirsutism evaluation and facilitating data comparison. The modified FG (mFG) score evaluates hair growth in nine body areas. Hair growth is rated from 0 (no terminal hair) to 4 (male pattern hair) in each area, with scores of 1, 2, and 3 indicating intermediate levels of body hair growth, for a maximum score of 36 (Figure 1). The original method had a cutoff of 5 to define hirsutism [41], which was increased to 8 in the mFG score, based on the original data published by Ferriman and Gallwey considering the 95th percentile [42]. In shaved areas, self-scoring can be clinically useful especially for follow-up, as it correlates only modestly with scoring by a trained observer [43].

**Figure 2:**
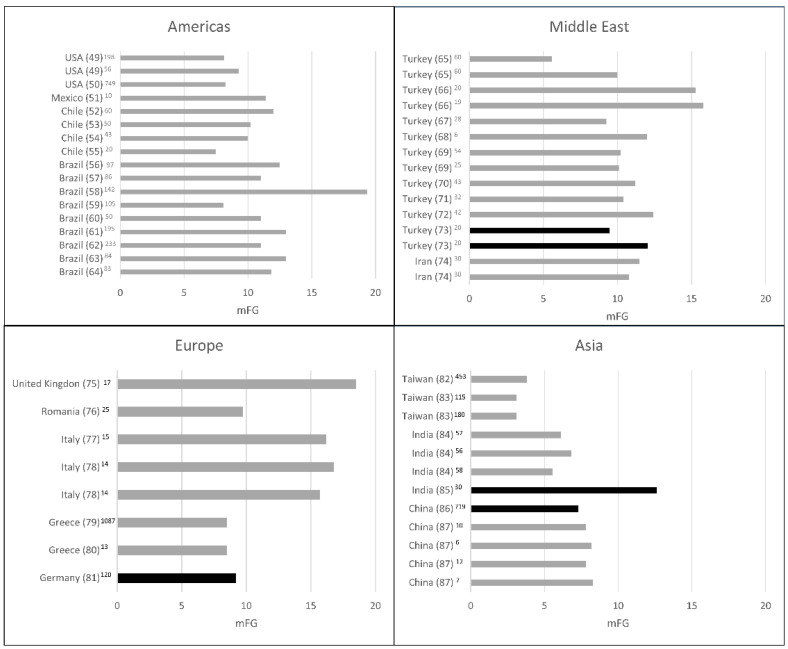
Hirsutism scores in women with polycystic ovary syndrome (PCOS) according to geographic distribution. PCOS was diagnosed by the Rotterdam, US National Institutes of Health (NIH), or Androgen Excess Society (AES) criteria. Black lines indicate unselected studies. Superscript numbers indicate sample size. mFG: modified Ferriman–Gallwey score.

Although a universal mFG score cutoff would be useful for comparisons, ethnic variations, as well as skin type and other factors, should be considered when evaluating hirsutism in distinct populations [44]. Importantly, the data reported by Ferriman and Gallwey were derived from women attending a northern London general medical outpatient clinic, a selected homogeneous population. Besides that, other factors, such as obesity and insulin resistance, are known to play a role in the phenotypic expression of PCOS, and its predisposition also varies in different ethnic backgrounds [45]. Indeed, the international evidence-based guideline for the assessment and management of PCOS, published in 2018, proposes to consider ethnic origin when evaluating mFG scores in different populations [1]. The Endocrine Society also suggests different cutoffs for the mFG score depending on ethnicity, as follows: United States and United Kingdom black or white women, ≥8; Mediterranean, Hispanic, and Middle Eastern women, ≥9 to ≥10; South American women, ≥6; and Asian women, a range of ≥2 for Han Chinese women to ≥7 for Southern Chinese women [46]. Subsequently, data from a systematic review comparing hirsutism in women with PCOS from populations of different ethnicities showed that, compared with white women, East Asian women were less hirsute, whereas Hispanic women, South Asian women and Middle Eastern women were more hirsute [47]. In another systematic review and meta-regression analysis, FG scores were presented according to their distribution in different countries [48]. However, few data are available on hirsutism in women with PCOS from Latin America. In this respect, Figure 2 shows a comprehensive distribution of mFG scores based on studies from different countries [49,50,51,52,53,54,55,56,57,58,59,60,61,62,63,64,65,66,67,68,69,70,71,72,73,74,75,76,77,78,79,80,81,82,83,84,85,86,87], which include available data from the database of a systematic review on Latin American women with PCOS that we have recently published [88]. Importantly, most studies come from referral populations, which may have influenced the difference in FG scoring between regions, according to ethnicities. 

In addition to ethnicity, skin type might be of some relevance to the hirsutism evaluation. The Fitzpatrick scale classifies skin type according to susceptibility to sunburn and melanin production in response to sunlight: type I (always burns, never tans), type II (usually burns, tans minimally), type III (sometimes burns mildly, tans uniformly), type IV (burns minimally, always tans well), type V (very rarely burns, tans very easily), and type VI (never burns, never tans) [89]. A study evaluating hirsutism according to the Fitzpatrick classification in 341 women (276 patients meeting the Rotterdam criteria for PCOS) found a significant difference in total mFG score and prevalence of hirsutism between different Fitzpatrick groups. Patients in group 3 (skin types V and VI) had the highest mFG scores and prevalence of hirsutism, followed by group 2 (skin types III and IV) and group 1 (skin types I and II). For the most part, the variation in hirsutism among skin types was due to differences in truncal mFG scores [90].

Age should also be considered when evaluating hirsutism, since it is well known that both clinical and biochemical characteristics change with age in women with PCOS [91,92,93,94]. Androgen secretion from ovaries and adrenal glands may diminish as a function of aging, and hirsutism scores accompany this trend [94]. In fact, hyperandrogenism may partially resolve before menopause in women with PCOS [93]. Serum concentrations of sex hormones increase from the pre- to postmenarchal periods as well [95], and mild hair growth is frequently seen in the late stages of puberty and early adolescence and may persist for several years; therefore, the diagnosis of PCOS is often not made until adulthood, when endocrine and metabolic dysfunctions have been firmly established [1,93]. Hence, ethnic origin, skin type, age and associated comorbidities (e.g., obesity and insulin resistance) must be combined when evaluating hirsutism in women with PCOS. Further studies with different populations may deeper clarify this issue and find practical ways to clinically evaluate hirsutism in distinct populations.

## 6. Diagnosing PCOS: Clinical versus Biochemical Androgen Excess

Measurements of biochemical parameters of hyperandrogenism are very useful, particularly in patients without obvious signs of clinical hyperandrogenism, such as hirsutism, acne vulgaris, and FPHL [96]. Hirsutism has a multifactorial pathogenesis that is mainly affected by androgen levels, with the sensitivity of hair follicles to androgens also playing a role in this process. In fact, the correlation of biochemical hyperandrogenism with hirsutism severity was found not to be as significant as expected, suggesting that there may be other factors contributing to the mechanism described above. In vitro studies have shown that both insulin and insulin-like growth factor-1 may also have a dose-dependent effect on hair follicle growth [97,98]. In fact, sex hormone-binding globulin, a modulator of testosterone bioavailability, is suppressed by hyperinsulinemia resulting from insulin resistance. Therefore, not only biochemical hyperandrogenism, but also insulin resistance may contribute to the development of hirsutism [99]. Data from a large sample of Korean volunteers showed that the homeostasis model assessment of insulin resistance (HOMA-IR) was positively associated with the FG score, even after adjustment for biochemical hyperandrogenism parameters [100]. Importantly, although within the reference range, patients with idiopathic hirsutism may have higher serum androgen levels and lower estradiol/testosterone ratio than healthy individuals, leading to relative hyperandrogenemia at the tissue level [101]. In addition, the response of the pilosebaceous unit to androgen varies considerably according to the skin area and between individuals, so not all patients with hirsutism have demonstrable hyperandrogenemia [102,103,104,105,106].

The biochemical assessment of sex steroids has important technical limitations. Androgen measurements in blood capture a moment in time, subject to the known pulsatility of these hormones [96]. Even in the setting of androgen excess, women usually show a slight increase in sex steroids, but the enzyme-linked immunosorbent or chemiluminescent assays used currently have low sensitivity and specificity in women. Indeed, the testosterone measurement may not be accurate at low levels (as expected in women), but liquid chromatography tandem-mass spectrometry, the most accurate method, is expensive and still not widely available [1,5]. Furthermore, serum androgen levels vary according to age, menstrual cycle day, and sampling time, but there is still no standardization based on these parameters [105]. Cross-reactivity between different steroids is another relevant issue related to the variability observed in androgen measurements in women [106]. In the clinical setting, only one-third of samples of patients with PCOS show abnormal testosterone, reaching 70% of samples for elevated serum concentration levels of free testosterone, the single most sensitive test for hyperandrogenemia [107]. Regarding other androgens, only 10% and 9% of patients with PCOS have isolated elevation of dehydroepiandrosterone sulfate (DHEAS) and androstenedione levels, respectively [108,109].

The concept of hyperandrogenism should be based primarily on clinical findings. Androgen measurements are not a substitute for clinical judgement, and it is particularly in patients without obvious signs of hyperandrogenism that biochemical evaluation is indispensable. Nonetheless, an abnormal scale of hirsutism associated with ovulatory dysfunction or ovarian morphological findings is sufficient for the diagnosis of PCOS, after the exclusion of related disorders as previously mentioned (96). The term idiopathic hirsutism should be applied only to hirsute women with normal ovulatory function and detectable normal androgen levels (testosterone, androstenedione, and DHEAS). In these cases, the cause of hair growth may be associated with abnormalities in peripheral androgen activity or increased sensitivity of the hair follicle [110].

## 7. Conclusions and Future Directions for Research

Several factors can potentially hinder the proper biochemical assessment of hyperandrogenism. Prominent among these are the questions of which androgens should be measured, which assay methods should be used, and how the reference ranges should be defined. Also of timely interest are the significant overlap of values obtained in women with PCOS and controls and the availability of and access to high-quality assays. Regarding clinical assessment, most patients with PCOS present with clinical features of hyperandrogenism, which are relatively inexpensive to assess and require only skilled vigilant clinicians. Both medical and cosmetic treatments of hirsutism have an impact on assessment.

## Data Availability

Not applicable.
